# Cross-Linguistic Similarity and Task Demands in Japanese-English Bilingual Processing

**DOI:** 10.1371/journal.pone.0072631

**Published:** 2013-08-28

**Authors:** David B. Allen, Kathy Conklin

**Affiliations:** School of English, University of Nottingham, Nottingham, United Kingdom; University of Leicester, United Kingdom

## Abstract

Even in languages that do not share script, bilinguals process cognates faster than matched noncognates in a range of tasks. The current research more fully explores what underpins the cognate ‘advantage’ in different script bilinguals (Japanese-English). To do this, instead of the more traditional binary cognate/noncognate distinction, the current study uses continuous measures of phonological and semantic overlap, L2 (second language) proficiency and lexical variables (e.g., frequency). An L2 picture naming (Experiment 1) revealed a significant interaction between phonological and semantic similarity and demonstrates that degree of overlap modulates naming times. In lexical decision (Experiment 2), increased phonological similarity (e.g., *bus*/basu/vs. *radio/*rajio/) lead to faster response times. Interestingly, increased semantic similarity slowed response times in lexical decision. The studies also indicate how L2 proficiency and lexical variables modulate L2 word processing. These findings are explained in terms of current models of bilingual lexical processing.

## Introduction

There is considerable evidence that cognates are processed more quickly than matched noncognates in a range of production (word naming: [1]; picture naming: [2]–[3]; word translation: [4]–[7]) and comprehension tasks (lexical decision: [8]–[9]; masked priming: [10]–[14]; progressive de-masking: [9]; sentence comprehension: [15]–[16]). Thus, the robustness of this cognate facilitation effect is attested across a wide range of tasks and with a number of first and second languages. The cognate advantage has been found even when languages do not share a script (e.g., Japanese-English, Korean-English, Hebrew-English, Greek-French).

Cognates share meaning (semantics; henceforth S) and form (phonological and/or orthographic; henceforth P and O) across languages. Their processing advantage could be underpinned by overlap in S, P, and/or O. The description of cognates in the psycholinguistic literature is usually based on the degree of overlap of O/P and S features across languages, instead of being described etymologically. Crucially, in the past the degree of O/P/S overlap has been used to select experimental materials, in other words, to decide whether a word was a cognate or not. More recently, a few bilingual studies have used continuous measures of similarity to explore how the amount of overlap influences processing of cognates and homographs [15]–[18]. However, this work has been done in languages that share a script, which means that the contribution of O and P overlap is hard to disentangle.

The current research investigates how cross-linguistic similarity influences bilinguals’ language processing in production and comprehension. The study provides the first evidence of how continuous measures of similarity can provide more comprehensive information about language co-activation in languages that differ in script and how this co-activation affects processing. In what follows we will first describe research on different script bilinguals, followed by a discussion of research using continuous variables of cross-linguistic overlap.

### Different Script Bilinguals

Recent work has shown that even for bilinguals whose languages differ in script (e.g., Japanese-English, Korean-English, Hebrew-English, Greek-French), cognate facilitation effects can be observed [12], [14], [19]–[21]. In a lexical decision task with Hebrew-English script bilinguals, Gollan et al. [12] found greater facilitation for cognates relative to noncognates when masked primes were in the L1 (first language) and targets in the L2 (second language). These effects were much weaker, however, when primes were in the L2 and targets were in the L1. Kim and Davis [19] explored whether priming occurred in three tasks (lexical decision, semantic categorization and word naming) for Korean-English bilinguals. L1 Korean primes facilitated recognition of L2 English cognates in all tasks, whereas noncognates facilitated responses in only the former two tasks, and homophones facilitated responses in lexical decision and naming only. Thus shared P and S similarity (without O similarity) appears to provide processing advantages for cognates in a variety of priming tasks, at least when primes are in the L1.

In a lexical decision task conducted using a masked priming paradigm [14], Greek-French bilinguals responded to L2 targets preceded by either related (translation) or unrelated (control) L1 primes. Voga and Grainger [14] found a priming effect of cognate translation primes relative to noncognate primes, indicating that L2 P information was activated by the L1 prime. Crucially, they also found that cognate targets that had high P overlap with their translation primes were responded to more quickly than to cognate targets that had low P overlap with their translations, when compared to noncognates. This finding shows that the degree of P overlap impacts the amount of cross-linguistic activation in lexical decision with masked translation priming. However, in Greek and English, there is some overlap in O (e.g., the cognates ‘kilo’ and ‘κιλó’ have three graphemes that are very similar), which makes it difficult to completely disentangle the influence of P and O in their priming effect.

In a lexical decision task, Taft [21] tested low proficiency Japanese-English bilinguals with two-syllable English words that were divided such that the coda or onset was maximized, (e.g., *ra dio* versus *rad io*). The items used in this study were either cognate or noncognate with English (i.e., they shared S and/or P features with English, but not O). Due to the influence of L1 Japanese, which typically has open syllables (*rad* cannot exist in Japanese, while *ra* can), participants responded more quickly to items such as *ra dio*, the maximal onset condition. Additionally, cognates were recognized significantly faster than noncognates (1118 ms versus 1186 ms), demonstrating the influence of P and S overlap from L1.

Finally, in a bilingual picture-naming task, Hoshino and Kroll [20] showed that the cognate facilitation effect is present in both same script (Spanish-English) and different script (Japanese-English) bilinguals. As picture naming does not involve the presentation of written words, cognate facilitation should be a product of the activation of similar P information across the two languages. P activation appears to be sufficient to create cognate facilitation in production for both same script and different script bilinguals. Importantly for the current research, these findings indicate that both of the languages of a bilingual are activated, even when the script is not shared. Further, cognates create greater cross-linguistic activation than matched controls.

### Degree of Similarity

In all of the aforementioned studies other than Voga and Grainger [14], experimental items were classified simply as cognate or noncognate (or homophone). However, the degree of similarity in both form and meaning varies greatly for translation equivalents; for example *bière, bier, beoir* in French, German, and Gaelic, respectively, can all be termed cognate with English *beer*. If overlap between words in two languages plays a role in cognate facilitation, it is important to assess the influence of the *degree* of overlap on facilitation. However, a weakness of many previous studies is that the methods used to determine ‘cognateness’ have often been unsatisfactory [22].

A study by Tokowicz et al. [22] demonstrated that raters are sensitive to the degree of formal similarity of Dutch-English translation pairs (in this case, sound-spelling cross-linguistic similarity). They showed that while many items were rated as having very little similarity (1–2 on a 7-point scale with 1 being ‘completely different’ and 7 being ‘identical’), raters also used the remainder of the scale (3–7) to differentiate between word pairs having differing degrees of formal similarity. Though this measure combined both O and P information in rating formal similarity, bilinguals rating languages with different scripts should be able to differentiate degree of formal similarity based on P alone.

Cognates are distinguished from other translation equivalents on the basis of shared formal features. However, both cognates and noncognates share some degree of S similarity with translation equivalents. Tokowicz et al. [22] also investigated cross-linguistic S similarity, hypothesizing that S similarity should be determined based on the number of shared senses and the similarity of these individual senses. They found that, while most word pairs (both cognate and noncognate) had high S similarity ratings, there was some variability across the items. They found that S similarity significantly correlated with the number of translations, context availability (the ease or difficulty of thinking of a context for a word), and concreteness measures: translation pairs that overall have fewer translations, that are more concrete and for which a context can easily be conceived are rated as more S similar. In sum, Tokowicz et al.’s [22] study suggests that S similarity is a useful theoretical construct for understanding bilinguals’ semantic representations and participants’ ratings are useful for establishing S overlap. Ratings can thus be used to define cognates objectively by setting a suitable threshold of formal and semantic similarity.

For languages that share script, some recent studies have examined how the degree of overlap influences processing. In a series of experiments Duyck et al. [18] manipulated the O similarity of words in a L2 (English) lexical decision task with Dutch-English bilinguals. They used orthographically identical and non-identical cognates and compared decision responses to matched control items. Cognate facilitation was observed for both identical and non-identical cognates in comparison to controls. A second experiment used a contextualized task where subjects read a visually presented sentence followed by a lexical decision task on the final word (the critical item). They found cognate facilitation for both types of cognates, although the cognate effect decreased when the words were not orthographically identical. Crucially, in this study the division between identical and non-identical cognates was binary. However, if the amount of overlap between languages modulates processing, then a more subtle manipulation will be needed to detect this.

In a rating study, Dijkstra et al. [17] had 24 Dutch-English bilinguals rate 360 words for O, P and S similarity. Unsurprisingly, they found that O and P ratings were highly correlated (*r* = .94, *p*<.001), meaning it is necessary to control for this correlation when assessing the individual influence of these characteristics. In a lexical decision task, they found that the ratings predicted responses times, such that that increased O similarity lead to faster responses to non-identical cognates, while P similarity had no influence. Moreover, for orthographically identical cognates there was increased facilitation when P similarity was greater, indicating that when O overlap is complete, P information becomes another source of information that is exploited. Another key finding of Dijkstra et al. [17] was that the direction of effects of P similarity depended on the task conditions. In both L2 lexical decision and progressive demasking tasks, when English targets were P similar but S dissimilar (homophonous) to Dutch words, they were responded to more slowly than controls. The influence of L1 in the L2 task thus provided evidence for non-selective activation in bilinguals’ processing of language, but importantly for the present study also provides evidence that P similar words can lead to inhibition of responses latencies under certain conditions.

The influence of cross-linguistic O overlap has also been shown for cognates when reading sentences in the first language [15]. Using Van Orden’s [23] measure of O similarity for Dutch-English word pairs, Van Assche et al. [15] showed that as O overlap increased, cognates were read more quickly and this effect did not differ depending on whether sentences were high or low constraint. In a more recent study, Van Assche et al. [16] demonstrated significant effects of both an objective measure of O overlap and a combined measure of O and P overlap on lexical decision times to Dutch-English cognates presented in the L2 (English). Similarly, in sentence reading both early and late measures of fixation duration showed facilitatory effects of overlap, which was not greatly affected by sentence constraint (high vs. low). However, given the high correlation between the O and P similarity ratings for Dutch-English cognates, it is difficult to assess the singular contribution of P similarity on bilingual word recognition.

For languages that differ in script, P similarity becomes the only measure of formal similarity, and can distinguish cognates from noncognates as well as provide a metric for degree of overlap for cognates. For example, the Japanese loanwords *bus* (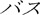 (/basu/) and *radio* (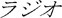/rajio/) can be classified as Japanese-English cognates because they share P and S features. Japanese words such as 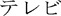/terebi/are accurately referred to as loanwords as they are borrowed into the language from English; however, in psycholinguist terms overlap and not the origin of the words is what is important, and thus in the paper these are referred to as cognates. However, *bus* in Japanese (/basu/) intuitively sounds more similar to its English equivalent, while *radio* sounds more distinct from/rajio/. Differences in phonotactics and the phonetic inventories of the two languages, contribute to the degree of P overlap in these cognate/borrowed words. Based on previous studies with same-script bilinguals [15]–[18], we expect that the degree of P overlap will modulate cognate facilitation in Japanese-English bilinguals. Because English and Japanese utilize different O scripts, no influence of O is expected. These predictions follow the theoretical assumptions of the revised Bilingual Interactive Activation Model (BIA+; [24]) for word recognition. In this model, O is presumed to be incapable of creating cross-linguistic effects in languages that differ in script. P cross-linguistic activation is predicted by the BIA+ in the absence of a shared O, and the degree of this cross-linguistic activation is dependent on the degree of P similarity of translations across languages. For language production, a similar prediction can be made based on models such as that proposed by Costa et al. [2]–[3] for picture naming. While this model has been described in terms of same-script bilinguals (Spanish-Catalan and Spanish-English), it is potentially applicable to different-script bilinguals because picture naming does not necessitate O activation in order to produce a response. Thus, in line with this model and in the absence of O, P similarity should be the key determiner of cross-linguistic activation via formal features, such that increased P overlap leads to faster responses in picture naming. The focus of this research is thus how L1 P, not O, influences processing in the L2.

In addition, S similarity is an important variable when assessing degree of overlap for cognates, but also varies for translation pairs [22]. Thus, we may see further modulation of cognate processing based on the degree of S similarity. Specifically, increased S similarity may be expected to speed responses in tasks that constrain semantic activation to one particular sense, such as picture naming. In this task, picture stimuli activate conceptual features that feed forward activation to the appropriate lexical representations in both languages that are associated with the picture (i.e., the pictures’ names). If the word has multiple senses (e.g., *bat* can refer to ‘the creature’ or ‘the sporting equipment’), the alternative senses may be activated via feedback from lexical representations to conceptual features, and this activation may cause competition between the different conceptual features. If this is the case then activation of multiple senses may be expected to slow responses in picture naming. Because items with high S similarity ratings tend to have few senses across languages and these are more likely to be shared, such items should be named more quickly than item with low S similarity.

In contrast, in tasks that do not constrain the activation of particular senses, having multiple senses may actually be an advantage. In a lexical decision task, when the word *bat* is presented, activation of either the meaning ‘creature’ or ‘sporting equipment’ can lead to the correct “Yes” response. Unlike in picture naming, activation of multiple meanings should not cause competition as all should lead to the same response. In previous research, words with multiple senses are responded to more quickly in lexical decision relative to those with few senses (e.g., [25]); however, words that have multiple senses that are highly distinct (e.g., *bank* in English) have been shown to lead to slower responses due to competition between these different senses [26]. In sum, depending on the number of senses of the stimuli, how related the senses are and the type of task, responses may be facilitated or inhibited. A similar pattern of results may be expected for bilingual tasks. Specifically, in lexical decision we may see that responses to words with less S similarity (as long as the decreased S overlap is not due to distinct senses of words) will be speeded relative to words that have greater S similarity. In picture naming on the other hand, where semantic information is constrained, we may expect to see facilitation for items that have greater S similarity.

Such predictions are in line with current models of bilingual processing, such as the picture naming model proposed by Costa et al. [2]–[3] and the BIA+ [24]. Costa et al.’s model for picture naming [2]–[3] assumes that multiple semantic nodes (conceptual nodes) become activated on recognition of the picture stimulus and that these nodes feed forward activation to lexical nodes. Thus, in picture naming, greater cross-linguistic S similarity would be advantageous. Increased shared conceptual features would lead to greater activation of both languages’ lexical nodes. Conversely, if a target in one language had multiple senses, one of which was appropriate for the target while others were not, activation of the inappropriate senses via feedback from lexical to semantic nodes could potentially create inhibition in naming.

While Costa et al.’s [2]–[3] model is specifically for picture naming, the BIA+ is specifically for word recognition and has a task/decision system that allows decision criteria to be modified depending on the task. The BIA+ would predict that in lexical decision, semantic activation is necessary to execute a correct response. This process does not require activation of a particular sense; rather any activated sense is sufficient to allow the correct response. When targets are presented that have multiple senses, the combined semantic activation of these senses deriving from lexical and sublexical activation during word recognition could actually speed responses relative to words that have a smaller number of senses. Thus, in lexical decision, when words have more senses in either or both languages (i.e., words with less S similarity), this should lead to facilitation.

### The Present Research

The aim of the present study is to investigate the role of P and S similarity in the processing of languages that differ in script, and to determine whether continuous measures of similarity can further illuminate cross-linguistic effects above and beyond binary cognate-noncognate classifications. To do this, we utilised mixed-effects modelling with multiple continuous measures and fitted a model for the data. To investigate the role of continuous measures of P and S similarity in both bilingual language production and recognition as well as the interaction between these two measures, we conducted two L2 tasks: picture-naming and lexical decision. Picture naming limits the types of words that can be explored (concrete), while lexical decision allows for the use of a range of words (concrete and abstract items, nouns and verbs). Thus, only a subset of the items in the lexical decision task is appropriate in the picture naming task. Because some of the items appear in both tasks, to avoid effects of repetition priming, two closely matched sets of bilinguals were tested in Experiments 1 and 2. In spite of these differences, the use of a production task and a lexical decision task allow us to explore how cross-linguistic measures might depend on different task demands. Namely, the role of P overlap and its potential interaction with S overlap may differ in production and comprehension tasks.

Although mixed-effects models do not necessitate matching items, as in typical factorial experiments testing cognates and noncognates, because we wish to maintain comparability with previous factorial studies, and simultaneously compare the effects of continuous similarity measures with binary measures of cognate status, we maintain the principle of item matching. Thus, while all items are initially distinguished by cognate status and matched accordingly, we can also add matched terms to the model to control for these effects more precisely.

## Rating Study: P and S Similarity

A rating study was conducted for the items used in Experiments 1 (picture naming) and Experiment 2 (lexical decision). Japanese-English bilinguals rated word pairs (e.g., *radio-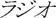
* (*/*rajio*/*) or *ear-* 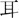
(/mimi/)) on a scale of 1 to 5 (1 = completely different, 5 = identical) for either P or S similarity. Because Japanese and English do not share a script, P similarity is crucial whereas O similarity should not play a role. Japanese cognates and noncognates are typically, but not always written in different scripts. Cognates are usually written in katakana and noncognates are written in any of the three scripts, but with kanji and hiragana being more common than katakana. The difference between the Japanese scripts typically used for cognates and noncognates is unimportant because none of the L1 Japanese scripts is based on the Roman alphabet. Thus there are no differences in O overlap with English and the Japanese scripts. Moreover, both tasks are entirely in L2 English, limiting any potential cross-linguistic O influence. Even in Experiment 2 (lexical decision), which involves L2 O, differences in L1 script should not matter. Cross-linguistic activation of formal features should be at the level of P only. Importantly, if L1 O codes are activated, feedback should not differentially influence L2 O processing, because none of the scripts overlap with the L2 O code. According to the BIA+ [24] different script languages do not have any cross-linguistic activation at the level of O. Nonetheless, because Japanese scripts do differ for cognates and noncognates, it is not possible to completely rule out the effect of L1 script on L2 processing. For the first set of 162 concrete items, 40 Japanese-English bilinguals rated half of the items for P and the other half of the items for S similarity, meaning that each item was rated 20 times for both P and S similarity. For the second set of 120 abstract items, a different group of 39 Japanese-English bilinguals similarly rated half of the items for P and half for S similarity. Because S similarity is likely to be reasonably high for all translation equivalents, 20 non-translation equivalent word pairs were added as filler items, to encourage raters to utilise all parts of the scale for both P and S similarity ratings. Including non-translations may reduce the focus on nuanced differences between translations. However, there are two reasons why this is unlikely be the case: firstly, there were only 20 non-translations included meaning their overall frequency was minimal in the task; secondly, the distribution of responses for both S and P similarity show that ratings varied across the scale indicating that nuanced differences in meaning and also difference in form were taken into consideration by raters. In both rating studies, cognates were rated as significantly more P similar than noncognates (concrete items: cognate M = 3.4, SD = 0.8; noncognate M = 1.01, SD = 0.02; *p<.*001; abstract items: cognate M = 3.4, SD = 0.6; noncognate M = 1.1, SD = 0.1; *p<.001*), while there was no difference for cognate and noncognates in terms of S similarity (concrete items: cognate M = 4.5, SD = 0.3; noncognate M = 4.4, SD = 0.4; *ns*; abstract items: cognate M = 4.3, SD = 0.4; noncognate M = 4.1, SD = 0.7; *ns*). Similar to Tokowicz et al. [22] we found that raters used the whole scale for rating P similarity. S similarity ratings for experimental items clustered at the ‘identical’ end of the scale but there was some variation in S similarity. As expected, the non-translation equivalent filler items were clustered at the opposite (‘completely different’) end of the scale (M = 1.2, SD = 0.1). The mean ratings of P and S similarity for items are used as the cross-linguistic similarity measures in the following experiments.

## Experiment 1: Picture Naming in L2 English

To test the effect of cross-linguistic similarity in language production with bilinguals whose languages differ in script, we performed an L2 picture-naming task making use of words that differed in their degree of cross-linguistic P and S similarity. The present study extends previous research [20], in which a cognate effect was found in L2 picture naming in different script bilinguals, by utilising continuous measures of similarity as well as by accounting directly for other factors (e.g., word length, frequency, and proficiency) in a mixed-effects model.

### Methods

#### Participants

Twenty participants (16 male; mean age = 20 y, ±3 y) from the University of Tokyo were paid for their participation. All participants were native Japanese speakers and had similar proficiency in English (see Table 1 for participant characteristics). All participants performed satisfactorily in the task and thus data from all participants is used in the analyses. All participants completed informed consent forms prior to participating in the research described in this paper. The University of Nottingham, School of English ethics committee, approved all studies reported in this paper.

**Table 1:** Participants’ characteristics in Experiments 1 and 2.

	Experiment 1		Experiment 1	
Proficiency (self-ratingfrom 0–10)	L1	L2	L1	L2
Reading	9.9 (0.5)	6.5 (1.3)	10 (0)	7.4 (1.2)
Writing	9.8 (0.7)	4.7 (1.7)	10 (0)	5.9 (1.7)
Speaking	10.0 (0.2)	3.8 (1.9)	10 (0)	4.4 (1.6)
Listening	10 (0)	5.5 (2.0)	10 (0)	6.2 (1.3)
Mean	9.9 (0.3)	5.1 (1.5)	10 (0)	6.0 (1.3)

#### Materials

Twenty-seven matched pairs of cognate and noncognate words were selected for the L2 English task (see Supporting Information for items used in both experiments: Table S1 and Table S2). The corresponding picture stimuli were from Székely et al. [27]. Cognate and noncognate items were matched on English word length, number of syllables, naming agreement (H statistic), mean naming latency, mean objective age of acquisition, mean conceptual familiarity, phonological neighbourhood size, phonological onset (fricative/non-fricative) and objective frequency. The data for the first six variables were taken from Székely et al. [27]. To account for the familiarity of the cognate and noncognate pictures, conceptual familiarity measures were taken from Nishimoto, Miyawaki, Ueda, and Une [28] who asked native Japanese speakers to rate how familiar they were with the concept depicted in pictures from Székely et al. [27]. Data on phonological neighborhood size was gained from the Elexicon project [29]. Finally, frequency measures were taken from the BNC (British National Corpus) including both the spoken and written components [30]. The frequencies are token frequencies taken from a total wordlist downloaded via the Sketch Engine website [31]. Japanese word frequencies are also token frequencies, and when multiple readings are used (i.e., any combination of kanji, hiragana and katakana) the summed total of each reading’s frequency is used. As Table 2 demonstrates, all of the cognate-noncognate pairs were matched as closely as possible on all of the variables, and there were no significant differences between cognates and noncognates on any of the variables, *p*’s>.1. In addition to the experimental items, thirty noncognate filler items were selected at random from the picture database [27] to reduce the overall frequency of cognates in the experiment. Twenty practice items (5 cognate, 15 noncognate) were also selected at random from the database. Pseudo-randomized lists were created to ensure that no two cognates and no words from the same semantic category or with the same phonological onset in English occurred in sequence.

**Table 2:** Stimuli characteristics for matched cognate and noncognate words in Experiment 1.

Variable	Cognate	Noncognate	*P* value (t-test)
Length	5.22	5.26	0.93
Number of syllables	1.63	1.48	0.47
Naming agreement	0.26	0.25	0.87
Word naming latencies (ms)	850.37	849.21	0.97
Age of acquisition (scale of 1–3)	1.81	2.04	0.41
L1 conceptual familiarity (scale of 1–7)	5.09	5.31	0.51
Phonological neighborhood size	10.6	10.5	0.97
Phonological onset (no onset fricative = 0, onset fricative = 1)	0.22	0.33	0.37
Frequency per million words (BNC)	4635.63	6564.33	0.33
Phonological similarity	3.47	1.08	<0.01
Semantic similarity	4.39	4.43	0.98

#### Procedure

Participants were tested in a quiet room. Both the instructions given on-screen and by the experimenter were in English. A language background questionnaire was completed following the experiment to assess language proficiency. The experiment was constructed using DMDX [32]. Participants were seated in front of a computer (Dell, English OS) connected to a headset. They sat around 40–50 cm away from the screen with eyes level with the centre of the screen and were instructed to name the picture as quickly and accurately as possible in English. They were told to refrain from using hesitation words and say ‘don’t know’ if they did not know the answer. Each trial began with a “+” fixation mark for 2000 ms followed by the picture stimuli at which point response timing began. Responses were detected using the headset’s microphone at which point the picture was removed and the following trial initiated. If no response was detected during 10000 ms of presentation, the following trial began automatically.

### Results and Discussion

Accurate responses were trimmed for outliers and errors. An accuracy analysis using a *X^2^* test of the number of errors for cognates (4.4% of total responses) and noncognates (6.0% of total responses) revealed no difference in terms of the number of accurate responses (*X^2^* = 0.005, df = 1, *p* = .94). This result may reflect the fact that items were equally familiar in both conditions. Correct responses that were less than 300 ms or greater than 3000 ms and outliers that were 2.5 standard deviations from the mean were removed from RT analyses (a further 6.9% of the total data). Items that had overall error rates of over 30% were removed along with their matched counterpart (8 items in total, half cognate and half noncognate). All false starts and ‘don’t know’ responses were classed as errors and removed. Minor deviations from the target name were allowed if they were extensions *forefinger* (for *finger*) or truncated forms of the target item *phone* (for *telephone*). Accepting deviations may introduce additional “noise” into the data due to differences in frequency and word length of experimental targets compared to the control ones. However the number of deviations in the present experiment was very small (1.3% of the total data) and critically, when these deviations were removed from the analyses the pattern of findings remained the same. This trimming of data resulted in a further 8.4% of the total data being removed bringing the complete percentage of data removed to 25.7%. The average response times and accuracy rates for both experiments are shown in Table 3 below. In picture naming, *t*-test comparisons revealed that neither accuracy or response latency were significantly different for cognates and noncognates (*p*<.05).

**Table 3:** Japanese-English bilinguals’ mean response latencies and error rates for Experiments 1 and 2.

	L2 picture naming			L2 lexical decision		
	Cognate	Noncognate	Difference	Cognate	Noncognate	Difference
Mean RT (SD)	1308 (524)	1362 (511)	54 ms	706 (214)	727 (200)	21 ms*
% Error	4.4%	6.0%	1.6%	5.6%	8.3%	2.7%*

Standard deviations are in parentheses; Asterisks indicate where paired t-test comparisons of cognates and noncognates were significant to *p*<.05.

To explore the contribution of the various factors, mixed-effects modelling [33] was conducted with R version 2.11.1 [34] and the R packages MASS, lme4, lattice and Design, and LMERConvenienceFunctions [35]. The following predictors were considered in the model: *Mean Phonological similarity* (PhonSim); *Mean semantic similarity* (SemSim); *Mean self-rated L2 proficiency* (Prof.av), which was calculated as a composite mean of four individually rated language skills (speaking, listening, reading and writing); *English word frequency* [30]; and *Japanese word frequency* [36]. Additional predictors included *word length* (Length), *conceptual familiarity* (JpFam) and *English objective age of acquisition* (EnAoA) as these have been shown to be significant predictors of picture naming in other studies using similar stimuli [27]–[28]. Two task-related predictors were included: Trial number (Trial), which has been shown to account for variance in responses attributable to practice effects and task fatigue [33], and Previous RT (PrevRT), which is a measure that uses the previous trial’s RT as a predictor for the current trial and has been successful at accounting for variance attributable to task factors [33]. Moreover, interactions between P/S similarity and L2 proficiency as well as L2 frequency were included. The response latencies and measures of *English* and *Japanese word Frequency* were log-transformed to increase normality and minimize random variance.

A correlation analysis was performed for all item predictors to ascertain which were significantly correlated. When two or more predictors were significantly correlated, this collinearity was removed by fitting a linear model in which one variable became the response and was predicted by the other correlated variables. For example, if word length was correlated with word frequency and P similarity then word length was used as the response variable in a model with word frequency and P similarity as predictors. Similar models were then made for the word frequency and P similarity as the response variables with all their correlated predictors (including previously residualized response variables, such as word length in the example). The residuals of these models were used as predictor variables in the final analyses. The resulting residuals were significantly correlated with their related variables (*p*<.01): log English Frequency (BNC_resid; *r* = .74), log Japanese frequency (AK_resid; *r* = .71), English AoA (EnAoA_resid; *r* = .90), English word length (Length_resid; *r* = .89), conceptual familiarity (JpFam_resid; *r* = .83), P similarity (PhonSim_resid; *r* = .92) and S similarity (SemSim_resid; *r* = .85). By-subjects random slopes for predictors tied to items and by-items random slopes for predictors tied to subjects were also fitted.

A backward simplification procedure was automated using the package LMERConvenienceFunctions [35], such that all terms and interactions were in the initial model and non-significant interactions and individual terms were removed step-by-step. Interaction terms were always removed prior to individual terms, and each time a term was removed an ANOVA (Analysis of Variance) and log-likelihood ratio testing was performed to test whether this removal significantly affected the predictive capability of the model. If the removal was significant (*p*<.05) then the term was retained in the model. The coefficients of the fixed effects, their Higher posterior Density (HPD) intervals, *p*-values based on 10,000 Markov Chain Monte Carlo samples of the posterior samples of the parameters of the final models and the *p*-values obtained from *t*-tests are presented in the final model for response latencies in the L2 picture-naming (Table 4). The standard deviation, median and mean coefficients based on MCMC sampling, and HPD intervals for random effects of participants and items in the final model are shown separately in Table 5.

**Table 4:** Final model for picture naming: Fixed effects.

	Estimate	MCMCmean	HPD95lower	HPD95upper	pMCMC	Pr(>|t|)
(Intercept)	7.165	7.163	7.097	7.242	0.001	0.000
cTrial	0.002	0.002	0.001	0.004	0.004	0.005
logBNC_resid	−0.001	−0.001	−0.031	0.031	0.948	0.943
Length_resid	0.040	0.040	0.015	0.065	0.001	0.002
JpFam_resid	−0.084	−0.083	−0.116	−0.048	0.001	0.000
PhonSim_resid	−0.022	−0.022	−0.050	0.003	0.088	0.136
SemSim_resid	−0.067	−0.065	−0.141	0.026	0.130	0.135
cProf.av	−0.075	−0.075	−0.123	−0.025	0.006	0.007
logBNC_resid:PhonSim_resid	−0.060	−0.059	−0.083	−0.036	0.001	0.000
PhonSim_resid:SemSim_resid	−0.073	−0.075	−0.138	−0.019	0.024	0.029

**Table 5:** Final model for picture naming: Random effects.

Groups	Std.Dev.	MCMCmedian	MCMCmean	HPD95lower	HPD95upper
Item (intercept)	0.092	0.083	0.084	0.052	0.112
Participants (intercept)	0.167	0.142	0.143	0.102	0.187
Residual	0.279	0.283	0.283	0.269	0.297

**Figure 1:**
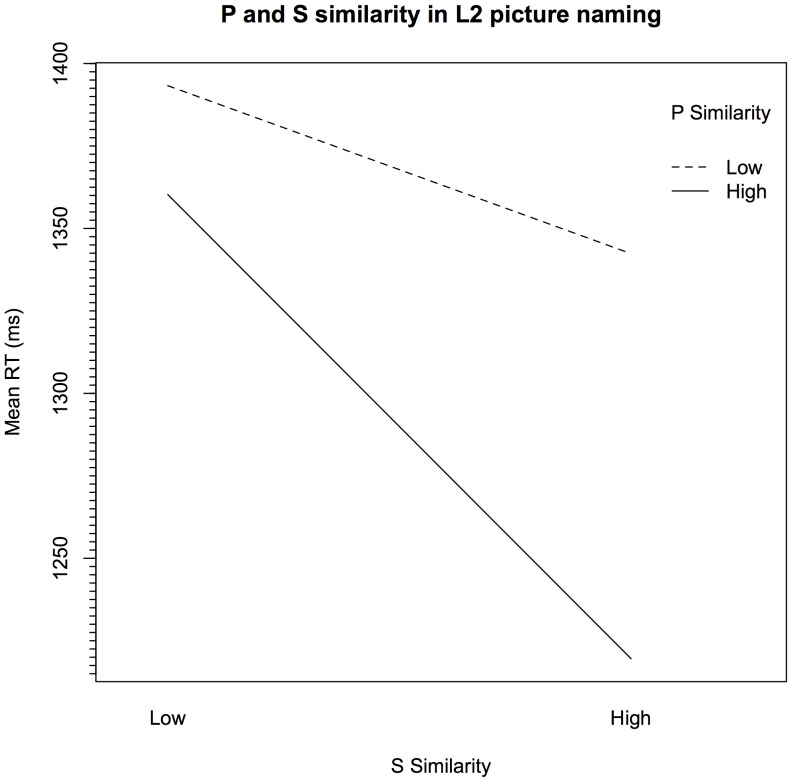
P and S similarity in L2 picture naming. For illustration purposes, S and P similarity ratings were divided into two equal groups along the median rating (Low, High).

Mixed-effects modeling showed that naming latencies were not significantly predicted by P similarity (*p*>.1). Also, S similarity was not a significant effect in the final model (*p*>.1). However, P similarity interacted with S similarity (*p*<.05), revealing an advantage for items that were both more phonologically and semantically similar across languages. This appears to show that it is the combination of both P and S similarity that lead to the ‘cognate effect’ as opposed to the contribution of the individual predictors. Figure 1 shows this effect clearly: responses to items with high P similarity ratings (i.e., those in the two highest quartiles) and increased S similarity are faster, whereas those with lower P similarity ratings (i.e., those in the two lowest quartiles) are less so. There was a 156 ms P similarity advantage for ‘high S similarity’ items (i.e., the difference between the highest and lowest RTs of this group), while there was only a 52 ms P similarity advantage for the ‘low S similarity’ items. This indicates that the combination of P and S similarity drives the cognate facilitation effect in picture naming.

**Figure 2:**
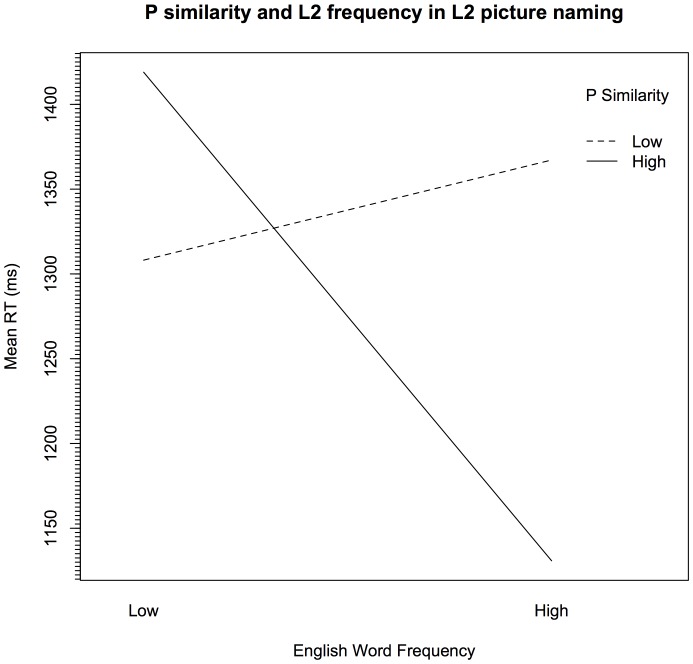
P similarity and L2 word frequency in L2 picture naming. For illustration purposes, P similarity ratings and log-transformed word frequency (taken from BNC [30]) were divided into two equal groups along the median rating (Low, High).

Moreover, another highly significant interaction occurred between P similarity and log-transformed L2 word frequency (*p*<.001). Responses to words with the greatest P overlap (cognates) were faster the higher their frequency (effect size = 288 ms). In contrast, items with the lowest P similarity (noncognates) appear to be slowed as a function of L2 frequency (effect size = 58 ms; Figure 2). We return to this in the General Discussion.

L2 proficiency was highly significant (*p*<.01), showing higher L2 proficiency speeds picture naming. Conceptual familiarity was a significant predictor of picture naming RTs (*p*<.01), with greater familiarity resulting in faster RTs. Length was also significant (*p<.*01), with longer words taking more time for participants to vocalize. Trial was significant (*p*<.05) and revealed an overall slowing of RTs during the course of the experiment, likely attributable to task fatigue. Alternatively this may be attributed to increased competition at the lexical level: as the task progresses more words become activated which creates greater competition for selection. We performed adjustments by including by-subject and by-item random slopes for predictor variables tied to items and subjects but none of these significantly improved the model (*p*<.05).

Above we have suggested that due to the variability in P similarity that cognate status in and of itself is less useful as an indicator of cross-linguistic similarity. However, like previous research (e.g., [20]), we can examine the binary cognate/noncognate status as a predictor of naming by classifying items as cognate or noncognate based on whether they are usually written in the katakana script (which is typically used for loanwords) or another script (i.e., kanji or hiragana, which are used for native and Sino-Japanese words). Thus, while the experiments reported in this paper do not involve presentation of words in Japanese, Japanese script information provides an unbiased way for differentiating loanwords/cognates from noncognates. Substituting P similarity with a binary cognate/noncognate classification, we found that cognate status was not a significant predictor in the final model (*p*>.1) but the interaction found between P and S similarity was replicated for cognate status and S similarity (*p*<.05).

To investigate whether the cross-linguistic similarity measures were sensitive to variation in responses to cognates, another analysis was performed using only cognate latencies. Mixed-effects modeling for the cognates revealed that RTs were shorter for words that had been rated as more P similar, though this difference was only marginally significant (*p*>.07). The effect for P similarity was larger when looking only at cognate items (estimate = −0.1544, *p*<.08), than when looking at both cognate and noncognate items together (estimate = −0.0224, *p*>.1). S similarity was highly predictive in the cognate-only model (*p*<.001), although it had not been significant in the full model (*p*>.1) with noncognates included. This indicates, as in the interaction in Figure 1, that S similarity had a greater effect when words were more P similar. The direction of this effect is negative, indicating that cognates with higher S similarity ratings were responded to more quickly than those with lower S similarity ratings.

To explain this finding the effect of S similarity it is necessary to consider the summed amount of activation that a lexical representation receives from conceptual activation via the picture stimulus. Considering a word such as *bat*, which has at least two distinct meanings, it is possible to assume that a picture stimulus of the animal *bat* would lead to activation of the lexical representation ‘bat’ and that little ambiguity exists as far as word selection is concerned. However, the fact that *bat* has multiple meanings may mean that alternative meanings are activated via feedback mechanisms from lexical to semantic representations. If this is the case, then multiple meanings may create a source of latent competition that influences word production. A related explanation that is applicable in the bilinguals’ case may be that words with multiple meanings in one language are more likely to multiple translations in another language [22]. Thus, *bat* may activate 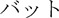/batto/“object for hitting” and 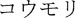/koumori/“animal” in Japanese. If this is the case, then competition may arise from the activation of multiple L1 translations. When words are more S similar and thus have fewer translations, this is likely to lead to less competition and thus faster responses, even when there exists no ambiguity as to the concept that is to be produced. This effect may be amplified for cognates relative to noncognates because of the influence of P similarity in activating one translation. If the picture is consistent with the activated translation, then faster responses may be expected (i.e. in the case of *(baseball) bat* and 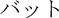). However, if the picture is inconsistent with the translation (i.e., in the case of *(baseball) bat* and 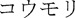/koumori/), then slower response times may be observed.

In sum, the findings of the present experiment provide a richer view of lexical processing in bilingual picture naming than previous studies. Due to the continuous nature of cross-linguistic similarity measures and the sensitivity of mixed-effects modeling to this, we get a more detailed picture of how overlap influences the production of cognates. Importantly, P and S similarity were not significant by themselves, indicating that they did not contribute over and above other lexical and semantic characteristics such as word frequency, length, age-of-acquisition or conceptual familiarity. Given the ubiquity of a processing advantage for cognates in languages that share a script, one might expect a clear influence of P similarity on cognate processing in the current study. However, findings for same script bilinguals cannot necessarily be applied to different script bilinguals. The current study, is to our knowledge, only the second to investigate the cognate advantage in picture naming with bilinguals whose languages differ in script. In the original study, Hoshino and Kroll [20] observed cognate facilitation. It is important to consider what might account for the difference between these two seemingly similar studies. The exclusion rate for data (errors, outliers and technical errors) in the current study was similar to that of Hoshino and Kroll [20], meaning that this is unlikely to account for the different pattern of results. The number of participants (*n* = 20) is smaller than in Hoshino and Kroll’s study (*n* = 27), but similar to other picture naming studies in the field ([37] *n* = 18; [38] *n* = 6 in an fMRI study; [39] *n* = 24). Thus, differences in the number of participants are unlikely to account for the different pattern of results. One reason for the difference may be that overall RTs were slower in the present picture-naming task than in Hoshino and Kroll [20], which allowed greater processing time thereby making the influences of cross-linguistic similarity more difficult to observe. Further, the Japanese-English participants in this research tended to be more proficient at reading than speaking (self-rated speaking M = 4.2, SD = 2.2; self-rated reading proficiency M = 7.2, SD = 1.2). Thus, it may be more likely to observe effects of cross-linguistic similarity in a comprehension task involving reading. In other words, because word recognition is faster than word production, the reduced time required between stimulus presentation and responses, as in a lexical decision task, may allow influences of cross-linguistic similarity to be clearly observed.

## Experiment 2: Lexical Decision in L2 English

In a picture-naming task the relationship between O and P overlap may be less important, as it primarily involves the activation of phonology. In a lexical decision task, both O and P are potentially important variables. However, previous research investigating the influence of degree of similarity on cross-language effects has made use of languages that share a script, making the role of O and P difficult to distinguish (e.g., *bière* and *beer* share both O and P). Because Japanese and English do not share orthography, they are ideal for exploring the contribution of meaning and form overlap, where form overlap is due to one variable, P, instead of two, O and P. Additionally, picture-naming limits the kinds of words that can be tested. Lexical decision task allows us to test whether P and S similarity measures were predictive of responses with a greater range of words (abstract and concrete). Finally, the use of different tasks will allow us to begin to explore whether the effects of cross-linguistic similarity might be dependent on task demands.

### Methods

#### Participants

Twenty-three participants (19 male, mean age = 19.9 y, SD = 5.1 y) from the University of Tokyo were paid for taking part in the study. All of the participants were native Japanese speakers and had a similar English proficiency (see Table 1 for participants’ language experience). Participants’ proficiencies were not matched across experiments and overall participants in Experiment 2 were higher proficiency. All participants performed satisfactorily in the task and thus data from all participants is used in the analyses. None of the participants had taken part in the rating studies or in Experiment 1.

#### Materials

Sixty cognates and 60 noncognates were selected and each group was made up of 30 concrete and 30 abstract words. Concreteness was established in a separate study where participants rated words on a 7-point scale (1 = abstract, 7 = concrete). Concrete words had a rating above 4.5 (M = 5.59, SD = 0.27) and abstract words had a rating below 4.5 (M = 3.22, SD = 0.63). Concrete items were selected from the same item pool as those in Experiment 1 (i.e., [27]), while abstract items were selected from a high-frequency wordlist derived from a 400 million Japanese web-corpus [31] to ensure all participants knew them. The cognates and noncognates were matched on a number of English characteristics: word length, average response time and accuracy, orthographic neighbourhood size, part-of-speech, word frequency, and concreteness. The first five of these measures were taken from the Elexicon database [29]; word frequencies were taken from the BNC [30]; and concreteness ratings were taken from the aforementioned rating study. As in Experiment 1, cognates and noncognates were matched as closely as possible with neither group being significantly different on any matched criterion (*p*’s>.1; Table 6).

**Table 6:** Stimuli characteristics for cognate and noncognate matched groups.

Variable	Cognate	Noncognate	*P* value(t-test)
Length	5.22	5.18	0.90
Number of syllables	1.55	1.55	1.00
Mean decisionlatencies (ms)	617	620	0.80
Mean decision accuracy	0.98	0.97	0.40
L1 concreteness (scale 1–7)	4.42	4.39	0.88
Orthographic neighborhoodsize	6.62	6.13	0.69
Number of senses:English	7.65	6.95	0.50
Number of senses:Japanese	1.83	2.27	0.20
Log frequency per millionwords (BNC)	7.72	7.57	0.60
Log frequency per millionwords (AK)	6.86	7.70	<0.01
Phonological similarity	3.42	1.11	<0.01
Semantic similarity	4.32	4.24	0.34
Part-of-Speech:			
Nouns	19	21	NA
Nouns/Verbs	30	22	NA
Verbs	0	2	NA
Adjectives	1	0	NA
Adj-Verb-Noun-Adverb	10	15	NA

In addition to the experimental items, 60 noncognate filler items were included to decrease the density of cognates in the experiment. One hundred and twenty nonwords were selected from the Elexicon database [29] and were matched with word items on length, orthographic neighbourhood size and average response accuracy. An additional 60 nonwords were selected to match the filler items on word length only. All nonwords were non-homophonic with Japanese words.

#### Procedure

Participants were tested in a quiet room. The language used in the on-screen instructions and in oral communication with the experimenter was English. Participants were seated in front of a computer (Dell, English OS) and responses were made via a keyboard press. The experiment was run using DMDX [32]. Subjects sat around 40–50 cm away from the screen with eyes level with the centre of the screen. Participants were asked whether they were right or left handed (of the 23 volunteers tested, only one was left-handed); “Yes” responses were always made with preferred hand. Participants were told to make word/nonwords responses; they were urged to respond as quickly and accurately as possible. Response times and accuracy were recorded automatically via keyboard presses. Stimuli were presented in lower case (Arial, size 14). Participants began the experiment by pressing the spacebar. A “+” fixation was displayed in the middle of the screen for 800 ms, followed by a black screen for 300 ms. Finally, a word or nonword appeared and remained on the screen for 5000 ms if no response was made. The next trial began immediately after a response was made or the trial timed out. Twenty practice trials preceded test trials and subjects were given feedback (i.e., “correct” or “incorrect” plus response time information) to encourage fast and accurate responses. No feedback was given during the experimental trials. Following the experiment, subjects completed a short survey detailing their language proficiency.

### Results and Discussion

For both analyses filler items and nonwords were removed. A *X^2^* test for count data shows that there were significantly fewer errors for cognates (5.6%) than for noncognates (8.3%; *X^2^* = 26.063, df = 1, *p*<.001), which is in line with previous findings in the literature (e.g., [19]). For the latency analysis errors (6.9% of responses) and outliers were removed. Outliers were responses falling ±2.5 standard deviations from the mean after errors had been removed and resulted in the loss of 4.6% of the data. The total proportion of data removed as errors and outliers was 11.5%. Mean correct RTs can be seen in Table 1, and were subjected to the same mixed-effects modelling procedure as Experiment 1. The predictors were the same except that conceptual familiarity and English-word AoA were not included, as these measures were only available for the concrete nouns.

The final model for response latencies is presented in Table 7 (fixed effects) and Table 8 (random effects). Unsurprisingly, trial number and previous RT are significant predictors of RTs (*p*<.001 and *p*<.05, respectively), showing that participants got faster at responding as the task progressed and that longer responses on previous trials led to longer responses on subsequent trials. English frequency was significant (logBNC, *p*<.001) but Japanese frequency was not (logAK, *p*>.1). English word length was significant such that longer words took longer to recognize (*p*<.001). Phonological similarity significantly speeded RTs (*p*<.01), indicating that L1 phonology is activated and facilitates recognition of L2 words. Greater semantic overlap significantly slowed RTs (*p*<.05), such that the greater the S overlap the slower the RTs. Potential reasons for this will be taken up in the General Discussion.

**Table 7:** Final model for lexical decision: Fixed effects.

	Estimate	MCMCmean	HPD95lower	HPD95upper	pMCMC	Pr(>|t|)
(Intercept)	6.542	6.542	6.492	6.593	0.001	0.000
cTrial	0.000	0.000	0.000	0.000	0.001	0.000
cPrevRTinv	0.009	0.009	0.002	0.016	0.010	0.010
logBNC_resid	−0.070	−0.070	−0.083	−0.056	0.001	0.000
Length_resid	0.067	0.067	0.055	0.079	0.001	0.000
PhonSim_resid	−0.024	−0.024	−0.035	−0.010	0.002	0.001
SemSim_resid	0.049	0.050	0.013	0.082	0.004	0.011

**Table 8:** Final model for lexical decision: Random effects.

Groups	Std.Dev.	MCMCmedian	MCMCmean	HPD95lower	HPD95upper
Item (intercept)	0.071	0.064	0.064	0.053	0.075
Participants (intercept)	0.145	0.119	0.121	0.092	0.153
Residual	0.198	0.200	0.200	0.194	0.205

As in Experiment 1, an additional analysis was conducted where P similarity was replaced by a binary cognate/noncognate variable. This yielded the same final model with similar effect sizes as the model with P similarity (cognate status estimate = −0.0577, *p*<.001). To explore whether P similarity simply serves as a proxy for cognate status, we further explored the role of P similarity in the set of cognate items. In the mixed-effects model with response latencies for cognates only, the P similarity measure was predictive of response times for cognates (estimate = −0.1035, *p*<.0.01), with increased P similarity leading to faster RTs. Again increased S similarity lead to slower RTs (estimate = 0.1542, *p*<.001). Both of these effects were larger than in the full model with cognates and noncognates, illustrating the role of P and S similarity variables as useful measures to explain bilingual performance when items are restricted to cognates.

In sum, Experiment 2 shows that P similarity ratings are predictive of RTs for words in the L2 and that subtle differences in cross-linguistic similarity have a significant influence on lexical decision speed. The finding that P similarity is significant for cognates (with noncognates removed from the analysis) suggests that subtle differences in P similarity across cognates leads to variation in processing speed, specifically that *more* P similar cognates are processed faster than *less* P similar cognates. P similarity thus illuminates cross-linguistic language processing effects above and beyond traditional binary distinctions of cognate status, as it determines processing speed of cognates relative to one another.

Interestingly, we find that S similarity is also predictive of decision responses, but with increased similarity resulting in slower decision times. To further investigate the locus of this effect we decided to include concreteness ratings (described previously) and the number of English senses (collected from WordNet [40]) as additional predictors in a post-hoc model for response latencies in lexical decision. If S similarity is predictive over and above these predictors (as well as those already included in the previous model, such as word frequency), then it can be assumed that S similarity accounts for cross-linguistic variation in responses that is not simply determined by the concreteness or number of senses that a word has in the target language (i.e., the L2, English). Correlated variables were dealt with using the procedure described previously and the residuals of these were used in the modelling process (i.e., conc_resid, ENoS_resid). Additional interactions between S similarity and concreteness, S similarity and English number of senses, and concreteness and English number of senses were also included in the initial model. The final mixed-effects model with concreteness and number of senses as additional predictors is shown in Table 9 (fixed effects) and Table 10 (random effects) below. Both additional predictors were highly significant (*p*<.001), such that increased concreteness led to slower RTs and increased number of senses led to faster RTs; moreover, S similarity remained significant (*p*<.01), revealing that S similarity does appear to predict variance in bilinguals’ responses that is not simply due to concreteness or number of senses.

**Table 9:** Final model with concreteness and number of English senses as additional predictors: Fixed effects.

	Estimate	MCMCmean	HPD95lower	HPD95upper	pMCMC	Pr(>|t|)
(Intercept)	6.531	6.531	6.477	6.583	0.001	0.000
cTrial	0.000	0.000	0.000	0.000	0.001	0.000
cPrevRTinv	0.009	0.009	0.002	0.015	0.002	0.009
logBNC_resid	−0.080	−0.078	−0.126	−0.035	0.002	0.001
logAK_resid	−0.006	−0.006	−0.017	0.006	0.294	0.297
Length_resid	0.073	0.073	0.061	0.086	0.001	0.000
PhonSim_resid	−0.025	−0.024	−0.038	−0.011	0.001	0.001
SemSim_resid	0.096	0.095	0.043	0.142	0.001	0.000
conc_resid	0.063	0.063	0.042	0.084	0.001	0.000
ENoS_resid	−0.015	−0.015	−0.020	−0.011	0.001	0.000
logBNC_resid:Prof.av	−0.008	−0.008	−0.015	−0.002	0.014	0.019
logAK_resid:PhonSim_resid	0.011	0.011	0.002	0.022	0.024	0.055
SemSim_resid:conc_resid	−0.042	−0.042	−0.074	−0.004	0.022	0.026

**Table 10:** Final model with concreteness and number of English senses as additional predictors: Random effects.

Groups	Std.Dev.	MCMCmedian	MCMCmean	HPD95lower	HPD95upper
Item (intercept)	0.069	0.063	0.063	0.052	0.075
Participants (intercept)	0.146	0.121	0.121	0.093	0.151
Residual	0.198	0.199	0.199	0.194	0.205

An interaction was also significant between S similarity and concreteness (*p*<.05), revealing that highly concrete items were responded to more quickly as S similarity increased, while words that were less concreteness (i.e., more abstract items) were responded to more quickly as S similarity decreased. An explanation for the latter may be found by considering the negative direction of the *number of senses* effect, which shows that items with more L2 senses are named faster than those with fewer senses: if words have a greater number of senses then these multiple senses may facilitate responses in lexical decision as shown in previous studies (e.g., [25]). Cross-linguistic S similarity is based on the number of senses and the number of these that are shared across languages, so it may be natural that S similarity and number of senses follow a similar pattern; however, we show here that both of these measures are significant. In sum, the findings from the lexical decision task show that concrete items are facilitated if they are more S similar across languages, while abstract words are instead facilitated by being less S similar across languages. Moreover, while S similarity and the number of English senses behave similarly, they appear to be at least partially independent.

Finally, a significant interaction was found between English word frequency and L2 proficiency (*p*<.05), such that the frequency effect was greater for bilinguals whose L2 proficiency was higher. This makes sense if we consider that as L2 proficiency increases, bilinguals are exposed to more English words and thus the subjective frequency of words also increases. This would lead to a larger L2 frequency effect for higher proficiency bilinguals.

## General Discussion

There is a large literature showing that cognates are processed more quickly than noncognates. However, most research to date has been conducted on languages that share the same script, and thus cognates in these languages overlap in O, P and S, which means that O and P overlap are often confounded. Two studies on Japanese-English cognate processing, where cognates share P and S but differ in O, demonstrate cognate facilitation in L2 picture naming [20] and in lexical decision [21]. These studies are important because they establish that shared O does not necessarily underpin cognate facilitation. However, because in these studies words are treated in a binary fashion, as cognates or noncognates, it is difficult to determine the potentially independent influence of P and S similarity on response times.

Experiment 1 showed that as the degree of cross-linguistic P similarity increases, *and* the degree of S similarity increases, words were produced faster. Thus, cognate items like *bus* (*/*basu*/*) and *radio* (*/*rajio*/*) were processed in English more quickly than noncognate items like *umbrella* (*/*kasa*/*) and *ashtray* (*/*haizara*/*), due to not only the degree of P similarity but also that of S similarity. However, the fact that the two similarity measures were not predictive as main effects suggests a limited role in word production, when accounting for other factors such as word frequency and word length. An alternative explanation for the lack of significant main effects in this experiment was the relatively slow responses overall to items, meaning that subtle influences of cross-linguistic similarity were less apparent. A replication of this study that includes a picture familiarization phase may help to speed up responses (as well as increase accuracy), leading to more observable cross-linguistic effects. Nevertheless, the interaction between P and S similarity observed in picture naming shows that bilinguals’ L1 was activated and influenced processing in the L2.

The interaction between P similarity and English word frequency raises the question as to why responses to cognates benefited from increased frequency, while noncognates were slowed by it. If L1 translations are activated by the picture stimuli, then there may be competition when the L1 and L2 translations do not share form (noncognates). In particular, when the L1 competitor is high frequency, competition may increase at the form level, which would slow naming times. When translations do share form (cognates), the L1 form does not compete for selection but instead increases activation of the L2 form. Therefore, increased L1 frequency increases L2 activation, thereby speeding naming times. Such a pattern may not have been observed before, because few regression-type designs have investigated bilingual picture naming studies. Moreover, it may be that the participants in the current study are highly L1 dominant, living in a relatively homogenous, monolingual community, which means that competition from L1 during L2 processing is more apparent than in previous research.

It may be unsurprising that in a naming task, where script is less likely to influence cross-linguistic activation, that we observe some influence of P and S on response times in different script bilinguals. However, in Experiment 2 where script could provide a strong cue for activation, we see that words having greater cross-linguistic P similarity were recognised faster and more accurately than those that were less similar. Importantly, P similarity discriminated between cognates, such that greater P similarity lead to faster RTs within the category of cognates. This shows that, although cognate status has typically been treated as a single category in previous studies, speed of processing is influenced by the amount of phonological overlap between the two languages. For example, *radio* is less phonologically similar to its Japanese translation (/rajio/) than *bus* (/basu/*)* is, and therefore *radio* is responded to more slowly (even after potential length effects have been accounted for). This result is in line with previous research showing that continuous measures of cross-linguistic similarity were predictive of RTs in bilingual tasks, but with same-script languages [15]–[16] and with a language where some of the script overlaps and some does not [14]. This indicates that P similarity can serve as a measure of formal similarity for languages that differ in script and that the amount of P similarity influences single word processing. We also see that increased S similarity leads to slower response times in lexical decision (this issue will be discussed below). Crucially, the current findings, with languages that do not share a script, add to a growing literature showing that it is more informative to use continuous measures of P and S similarity than using binary categories (cognate/noncognate), due to the inherent variability of words along these two criteria (cf. [17], [22]).

The influence of P similarity on L2 RTs in a lexical decision task suggests a strong influence of P in word recognition. There has been a long-running debate about the role of P in skilled readers’ word recognition processes, specifically whether P information is activated during word recognition or whether skilled readers by-pass activation of P representations and instead utilise a direct route from O to S representations (e.g., [41]). In many studies O and P are confounded because the languages under investigation share a script. Because Japanese and English do not share a script we can investigate the role of P overlap without an influence of O (a similar situation arises for other language pairs such as Korean and English e.g., [19]). The present research suggests a strong influence of P information in word processing, such that activation of L2 P information activates L1 word representations, and with increased P overlap there is faster word processing in the L2. Thus, for bilinguals with languages that differ in script, P information is not only sufficient to create cross-linguistic activation [20], but is critical in determining to what degree translation equivalents are activated in word recognition.

Interestingly, S similarity *speeded* response time in picture naming, at least in the interaction with P similarity and in the cognates only model, and *slowed* response time in lexical decision. This may be explained by task differences in Experiments 1 and 2. In lexical decision, activation of multiple meanings of words all lead to the same response, whereas a picture activates a particular word meaning and activation of alternative meanings may create competition during the word selection process. Previously with lexical decision it has been shown that words that have a greater number of meanings are recognised faster (e.g., [25]), presumably because the activation of multiple conceptual representations increases the activation of the lexical representation. In the current study, ratings of S similarity relate to the number of meanings shared across languages, such that words with more meanings have lower S similarity because fewer senses are shared. Using Wordnet [40] to count the number of English senses for the words in the current studies, we found that the number of senses is a significant predictor of S similarity: less S similar words have more individual senses. Because decreased S similarity indicates more meanings, our results are in line with findings showing that activation of multiple conceptual representations speeds lexical decision times. Moreover, the analysis including English number of senses and concreteness supports the idea that multiple senses speed responses in lexical decision, but importantly also shows that S similarity adds significantly to the model. Therefore, both expert defined number of senses (as in WordNet) and S similarity ratings from bilinguals appear to be useful measures of bilingual performance, even once collinearity is removed through residualization.

It is important to keep in mind that picture naming and lexical decision typically use different stimuli, as picture naming is limited to depictable, usually concrete words, while lexical decision can include both concrete and abstract words. Thus, lexical decision tasks can investigate a wide variety of words that differ in terms of S similarity, making it easier to explore the role of S similarity in word processing. Our study takes advantage of the fact that lexical decision can be used to investigate the processing of a greater range of words. Thus, while the two studies are not directly comparable because the picture naming task was limited to concrete words and lexical decision task investigated both concrete words (like in the picture naming task) and abstract words, taken together our results indicate that the direction of S similarity effects are dependent on task demands, but potentially also stimulus composition, with increased S similarity leading to speeded responses in picture naming but slower responses in lexical decision. This provides further evidence to move towards increasing specification of S features of stimuli as opposed to binary classifications of cognates and noncognates.

In Experiment 1 there was a clear contribution of proficiency, but proficiency was not predictive of RTs in Experiment 2. This discrepancy may be due to difference in the participants’ proficiency for speaking versus reading. In the present study, self-rated proficiency for reading considerably exceeded that for speaking (Experiment 1: reading M = 6.5 (SD = 1.3); speaking M = 3.8 (SD = 1.9); Experiment 2: reading M = 7.4 (SD = 1.2); speaking M = 4.4 (SD = 1.6)). The greater standard deviation for speaking in Experiment 1 suggests a wider range of proficiencies for production while that of reading in Experiment 2 suggests a smaller range for comprehension. Because Japanese learners of English must pass university entrance exams that do not include a speaking element, the focus in pre-tertiary education is on English comprehension. Thus, learners’ spoken fluency is more varied and often depends on extra-curricular experience such as studying abroad or attending conversation courses. Therefore, Japanese-English bilinguals typically, and more importantly in the present study, can be said to have more uniform L2 reading comprehension abilities in comparison to L2 production abilities. This uniformity, as well as the higher overall reading comprehension skills, may explain why there was no observed effect of proficiency in lexical decision.

One concern is that the L2 proficiency difference across experiments is responsible for the difference in the observed cross-linguistic similarity effects. Language proficiency is an important factor when looking at cross-linguistic influences, with unbalanced bilinguals (lower L2 proficiency, higher L1 proficiency) showing typically greater L1 influences in L2 processing (e.g., [11]). However, this means that it should have been more likely to observe a cognate effect in Experiment 1 than in Experiment 2, because participants in the first experiment had a lower L2 proficiency. Because proficiency was not matched across experiments, it is not possible to rule out the possibility that the different pattern of results across the two experiments is due to the proficiency of the participant groups. Investigating the role of proficiency and its potential interaction with continuous measures of P and S overlap with different script bilinguals in both production and comprehension tasks is an interesting question for future research.

Conceptual familiarity was highly predictive in picture naming, while word frequency was highly predictive in lexical decision. It is unsurprising that conceptual frequency is a predictor of naming latency for concrete images. Equally, it is not remarkable that word-based frequencies from written corpora are a more accurate predictor of written word recognition.

The modulatory function of P similarity for Japanese-English processing can be discussed in terms of interactive activation models of language processing. Costa et al. [3] discussed the results of a Spanish-English bilingual picture naming task in which P similar cognates were named faster than non-P similar noncognate controls. In their model of picture naming processing ([3], p.101), shared features at both the S node and P node levels create cross-language facilitation effects. This model is compatible with the present results, which in turn clarify that the *number* of shared features (i.e., the *degree* of similarity) at both of these levels influences naming and that this effect can be quantified using mixed-effects modelling. Because picture naming does not directly involve any processing of script, the findings and the model are compatible for languages that share and differ in script. For word recognition, the revised Bilingual Interactive Activation model (BIA+; [24]) can explain the present findings. The BIA+ model proposes that all words (in both of the bilingual’s languages) that share P and/or O features with the input become activated during the word recognition process. Residual activation of activated words feeds backward to the target item due to formal overlap of these items, increasing the activation of the target. Because Japanese and English do not share O, cross-linguistic activation is restricted to P similarity. The model predicts that as the number of shared P features increases, there should be increased feedback for the cognates, which can account for the current pattern of results. The S similarity measure in lexical decision reveals that words with more meanings are recognised faster. This is likely due to the task requirements of lexical decision where any activated meaning of a word sends activation back to the target, resulting in a negative relationship between number of senses and response speed. Thus, the current findings can be explained by a combination of activation of shared features and task demands within the BIA+ model.

The present study has demonstrated that a continuous measure of P similarity is a significant predictor of cross-linguistic activation and crucially that increased P similarity results in faster responses in L2 comprehension and also (in combination with S similarity) in production. A continuous measure of S similarity predicts response times and may be used, together with P similarity as a measure of ‘cognateness’ in languages that do not share a script. Importantly, using continuous measures of P and S similarity while controlling for other participant and lexical factors gives us a more complete picture of the role of cross-linguistic similarity on bilingual language processing than the more traditional binary distinctions.

## Supporting Information

Table S1 Target items used in Experiment 1 (depicted in pictures from [27]–[28]; 27 cognates, 27 noncognates).
(DOCX)

Table S2 Target items and matched nonwords used in Experiment 2 (60 cognates, 60 noncognates, 120 nonwords).
(DOCX)
