# Supplementary material for: Cross-Linguistic Similarity and Task Demands in Japanese-English Bilingual Processing
Source: PLoS One. 2013 Aug 28;8(8):e72631. doi: 10.1371/journal.pone.0072631 (PMC3755975; doi:10.1371/journal.pone.0072631)
Supplement: Table S1 — Target items used in Experiment 1 (depicted in pictures from [27]–[28]; 27 cognates, 27 noncognates). (DOCX) [file pone.0072631.s001.docx]

| English Name | Japanese name | Alphabetic transcription |
| --- | --- | --- |
| Banana | バナナ | banana |
| Bed | ベッド | beddo |
| Belt | ベルト | beruto |
| Bench | ベンチ | benchi |
| Brush | ブラシ | burashi |
| Bus | バス | basu |
| Button | ボタン | botan |
| Cake | ケーキ | keeki |
| Door | ドア | doa |
| Dress | ドレス | doresu |
| Fork | フォーク | fooku |
| Hanger | ハンガー | hangaa |
| Heart | ハート | haato |
| Helmet | ヘルメット | herumetto |
| Iron | アイロン | airon |
| Kangaroo | カンガルー | kangaruu |
| Lion | ライオン | raion |
| Pool | プール | puuru |
| Radio | ラジオ | rajio |
| Spoon | スプーン | supuun |
| Television | テレビ | terebi |
| Tent | テント | tento |
| Toaster | トースター | toosutaa |
| Truck | トラック | torakku |
| Trumpet | トランペット | toranpetto |
| Vest | ベスト | besuto |
| Yacht | ヨット | yotto |
| Car | 車 | kuruma |
| Cherry | 桜ん坊 | sakuranbo |
| Church | 教会 | kyoukai |
| Dog | 犬 | inu |
| Dolphin | イルカ | iruka |
| Elephant | 象 | zou |
| Finger | 人差し指 | hitosashiyubi |
| Frog | 蛙 | kaeru |
| Goat | ヤギ | yagi |
| Mirror | 鏡 | kagami |
| Moon | 月 | tsuki |
| Mountain | 山 | yama |
| Mouse | ネズミ | nezumi |
| Pencil | 鉛筆 | enpitsu |
| Plate | 皿 | sara |
| Rabbit | ウサギ | usagi |
| Rose | バラ | bara |
| Shoe | 靴 | kutsu |
| Snake | 蛇 | hebi |
| Sun | 太陽 | taiyou |
| Swan | 白鳥 | hakuchou |
| Telephone | 電話 | denwa |
| Train | 電車 | densha |
| Watch | 腕時計 | udedokei |
| Wheel | 車輪 | sharin |
| Window | 窓 | mado |
| Zebra | シマウマ | shimauma |
