# Supplementary material for: Cross-Linguistic Similarity and Task Demands in Japanese-English Bilingual Processing
Source: PLoS One. 2013 Aug 28;8(8):e72631. doi: 10.1371/journal.pone.0072631 (PMC3755975; doi:10.1371/journal.pone.0072631)
Supplement: Table S2 — Target items and matched nonwords used in Experiment 2 (60 cognates, 60 noncognates, 120 nonwords). (DOCX) [file pone.0072631.s002.docx]

| English name | Japanese name | Alphabetic transcription | Matched nonword |
| --- | --- | --- | --- |
| access | アクセス | akusesu | hieces |
| banana | バナナ | banana | bofied |
| bed | ベッド | beddo | tet |
| brush | ブラッシ | burashi | tomey |
| bus | バス | basu | zat |
| button | ボタン | botan | boofed |
| cake | ケーキ | keeki | mest |
| care | ケア | kea | pahe |
| career | キャリア | kyariaa | parbed |
| case | ケース | keesu | vare |
| classic | クラシック | kurashikku | sgrotch |
| cool | クール | kuuru | ceat |
| core | コア | koa | sare |
| course | コース | koosu | haples |
| cross | クロス | kurosu | ewact |
| curtain | カーテン | kaaten | topmyst |
| cycle | サイクル | saikuru | rynic |
| flute | フルート | furuuto | grues |
| fork | フォーク | fooku | tham |
| guitar | ギター | gitaa | ellnog |
| hammock | ハンモック | hanmokku | sioneer |
| hanger | ハンガー | hangaa | dester |
| helicopter | ヘリコプター | herikoputaa | spleatened |
| iron | アイロン | airon | erds |
| joke | ジョーク | jooku | vock |
| kangaroo | カンガルー | kangaruu | speories |
| kick | キック | kikku | pome |
| kiss | キス | kisu | yops |
| lion | ライオン | raion | jite |
| loan | ローン | roon | bood |
| local | ローカル | rookaru | nello |
| lucky | ラッキー | rakkii | sazer |
| necklace | ネクレス | nekuresu | flinness |
| pelican | ペリカン | perikan | blereof |
| penguin | ペンギン | pengin | pludies |
| pipe | パイプ | paipu | dutt |
| pool | プール | puuru | tove |
| pyramid | ピラミッド | piramiddo | pripend |
| race | レース | reesu | runk |
| rank | ランク | ranku | lage |
| regular | レギュラー | regyuraa | shafpud |
| rule | ルール | ruuru | lonk |
| scale | スケール | sukeeru | pords |
| score | スコア | sukoa | kacks |
| sense | センス | sensu | guels |
| show | ショー | shoo | goll |
| single | シングル | shinguru | scacks |
| size | サイズ | saizu | furg |
| ski | スキー | sukii | efa |
| skill | スキル | sukiru | shord |
| skirt | スカート | sukaato | toofs |
| slipper | スリッパ | surippa | glayling |
| slow | スロー | suroo | nent |
| style | スタイル | sutairu | ploss |
| tent | テント | tento | wast |
| tomato | トマト | tomato | ettcup |
| tractor | トラクター | torakutaa | partiam |
| trumpet | トランペット | toranpetto | sishful |
| violin | バイオリン | bairorin | clomax |
| work | ワーク | waaku | dran |
| acid | 酸 | san | boik |
| arm | 腕 | ude | olb |
| ashtray | 灰皿 | haizara | schiped |
| bicycle | 自転車 | jitensha | theppes |
| bone | 骨 | hone | sart |
| bricks | レンガ | renga | grajer |
| bust | 胸 | mune | lole |
| carrot | 人参 | ninjin | lelles |
| caution | 注意 | chuui | pimplos |
| chimney | エントツ | entotsu | clermos |
| clue | 手がかり | tegakari | ners |
| coral | サンゴ | saigo | atolp |
| cow | 牛 | ushi | bem |
| crime | 犯罪 | hanzai | halms |
| cure | 治る | naoru | mive |
| deer | シカ | shika | luty |
| demand | 要求 | youkyuu | lehind |
| dolphin | イルカ | iruka | pliffen |
| dresser | たんす | tansu | doasted |
| elephant | 象 | zou | alvisers |
| excited | 興奮 | koufun | truckeb |
| exit | 出口 | deguchi | flis |
| fail | 失敗 | shippai | poot |
| find | 見つける | mitsukeru | dall |
| firm | 会社 | kaisha | tuny |
| fish | 魚 | sakana | reag |
| front | 前 | mae | cleot |
| future | 将来 | shourai | peings |
| giraffe | キリン | kirin | blerved |
| hate | 憎む | nikumu | sile |
| ideal | 理想 | risou | aonta |
| joint | 関節 | kansetsu | chost |
| jury | 陪審 | baishin | jeed |
| learn | 習う | narau | efter |
| left | 左 | hidari | gour |
| lips | 唇 | kuchibiru | tave |
| lobster | ザリガニ | zarigani | ouplaws |
| loose | 緩い | yurui | cetty |
| matter | 物事 | monogoto | dacked |
| nose | 鼻 | hana | lote |
| past | 過去 | kako | gare |
| pencil | 鉛筆 | enpitsu | essigy |
| pig | 豚 | buta | fot |
| plain | 明白 | meihaku | bocer |
| prison | 刑務所 | keimusho | mailef |
| profit | 利益 | rieki | veware |
| sailor | 水兵 | suihei | mibing |
| scissors | はさみ | hasami | brylized |
| smell | 香り | kaori | freen |
| snake | ヘビ | hebi | forry |
| snowman | 雪だるま | yukidaruma | biewers |
| sock | 靴下 | kutsushita | zear |
| solid | 固体 | kotai | afoub |
| tank | 戦車 | sensha | fime |
| tiger | トラ | tora | tunch |
| trap | ワナ | wana | juff |
| turtle | カメ | kame | oubing |
| umbrella | 傘 | kasa | sulfido |
| view | 眺め | nagame | haip |
| warm | 暖かい | atatakai | kime |
